# Work-related burnout and its associated factors among midwives working at public hospitals in northwest Ethiopia: a multi-centered study

**DOI:** 10.3389/fpsyt.2023.1256063

**Published:** 2023-12-14

**Authors:** Berihun Agegn Mengistie, Zelalem Nigussie Azene, Tsion Tadesse Haile, Saron Abeje Abiy, Marta Yimam Abegaz, Eden Bishaw Taye, Haymanot Nigatu Alemu, Muluken Demeke, Mihret Melese, Nuhamin Tesfa Tsega, Getie Mihret Aragaw

**Affiliations:** ^1^Department of General Midwifery, School of Midwifery, College of Medicine and Health Sciences, University of Gondar, Gondar, Ethiopia; ^2^Department of Women’s and Family Health, School of Midwifery, College of Medicine and Health Sciences, University of Gondar, Gondar, Ethiopia; ^3^Department of Clinical Midwifery, School of Midwifery, College of Medicine and Health Sciences, University of Gondar, Gondar, Ethiopia; ^4^Department of Midwifery, College of Medicine and Health Sciences, Dilla University, Dilla, Ethiopia; ^5^The Department of Human Physiology, School of Medicine, College of Medicine and Health Science, University of Gondar, Gondar, Ethiopia

**Keywords:** Ethiopia, midwives, public hospitals, work-related burnout, burnout

## Abstract

**Introduction:**

Work-related burnout (WRB) is defined as the degree of physical and psychological fatigue and exhaustion that is perceived by the person as related to work. Midwives are vulnerable to work-related burnout due to their physically and emotionally demanding nature of their job. It affects the health of professionals and the quality of care provided. However, there is limited evidence on the burden and predictors associated with work-related burnout among midwives in developing countries, including Ethiopia. This study investigated the burden and contributing factors of work-related burnout among midwives in northwest Ethiopia.

**Methods:**

A facility-based cross-sectional study was conducted from February 7 to April 30, 2022. A simple random sampling method was used to enroll 640 study participants. The Copenhagen burnout inventory tool was used to assess the magnitude of work-related burnout. A self-administered questionnaire was used to collect data, which was then entered into Epi Data 4.6 software and exported to SPSS version 25 for analysis. A multivariable logistic regression analysis model was fitted to identify factors associated with work-related burnout. The Adjusted Odds Ratio (AOR) with 95% confidence interval (CI) was reported to declare the factors that are significantly associated with work-related burnout.

**Results:**

The prevalence of work-related burnout was found to be 60.47% (95% CI = 56.6–64.2). Workplace violence (AOR = 3.33, CI: 2.02, 5.48), working hours over 60 h a week (AOR = 4.55, CI: 2.78, 7.43), emotional demand of the job (AOR = 8.85, 95% CI: 4.48, 17.47), exposure to blood and body fluids/sharp injuries (AOR = 5.13, CI: 3.12, 7.13), good superior support (AOR = 0.38, CI: 0.23, 0.63), Job rotation of ≤6 months (AOR = 2.30, CI: 1.28, 4.14) and being stressed (AOR = 2.64, CI: 1.63, 4.26) were all found to be strongly linked to work-related burnout.

**Conclusion and recommendation:**

This study found a significant level of work-related burnout among midwives working in public hospitals. Experiencing workplace violence, a job rotation of less than or equals to six months, working hours over 60 h a week, good superior support, exposure to blood and body fluids or needle stick injuries and experiencing stress were significant factors that influenced work-related burnout. Therefore, reducing prolonged working hours, promoting supportive management, creating a safe working environment, and applying effective stress prevention strategies are some of the interventions to prevent or alleviate work-related burnout.

## Introduction

Although there are many ways to define burnout, the most popular is “a state of physical, emotional, and mental exhaustion that results from long-term involvement in work situations that are emotionally demanding (1).” “Work-related burnout (WRB) is defined as the degree of physical and psychological fatigue and exhaustion that is perceived by the person as related to his or her work (2).” Maternity care is one of the most essential sectors of the healthcare system, vitally contributing to the present and future health of society (3). Human resource personnel, particularly healthcare professionals, are at a significant risk of suffering burnout because of the stressful nature of their job (4). Evidence shows that midwives are at a greater risk of burnout than those in other professions (5). In particular, the work of midwives is emotionally demanding due to frequently facing an overwhelming workload, a shortage of resources in maternal health services, pain, anxiety, fear, stress, and emotional fatigue, experiencing traumatic events, or emotionally challenging circumstances such as workplace violence, and being underpaid for their work (6–9). A recent systematic review and meta-analysis study on burnout in midwives, out of 10 studies involving 5,946 midwives, reported a pooled prevalence of 40% work-related burnout (5). Besides, a significant level of exhaustion and fatigue was reported as 51% and 82.2% work-related burnout among midwives in Australia and Jordan, respectively (10, 11).

There are different validated instruments available for measuring burnout, including the Copenhagen Burnout Inventory (CBI) (2), and the Maslach Burnout Inventory (MBI) (12). The MBI is one of the most commonly used measurement scales in the literature, with a three-dimensional concept (emotional exhaustion, depersonalization, and low personal achievement) (12). The CBI is the other well-known measuring tool that comprises three subscales: personal-burnout, work-related burnout, and client-related burnout (2). Burnout is a chronic stress response that affects workers in a variety of workplaces (13). It’s characterized by both physical and mental exhaustion as a result of prolonged stress without effective coping strategies (13). Occupational stress is a pattern of negative and detrimental features of work content, work structure, and the working environment that results in emotional, cognitive, behavioral, and physiological reactions (14). Many mental and behavioral illnesses, such as burnout, depression, anxiety, and weariness, can be brought on by occupational stress (15). Occupation-related stress is a complicated notion since it encompasses both individual and specific labor activity elements. It can be associated with nature of working environment (crowdedness, temperature, ventilation, and lighting), organizational role, career growth, and various job tasks (shift works, night duties, heavy workloads, being exposed to risks and hazards, and being bored at work) (16).

Work-related burnout is an occupational phenomenon that is associated with negative consequences such as reduced productivity, job dissatisfaction, professional disengagement, higher risk medical errors, increased sick leaves, presenteeism, irritability, digestive disorders, undesirable patient outcomes, subsequent staff turnover, and a higher intention to leave a job (15, 17–21). In addition, it severely degrades the standard of treatment and diminishes client satisfaction while also leading to contempt and abuse of women during childbirth (18, 22, 23). As different literatures have demonstrated, burnout is multifactorial in nature (24, 25). Job stress, conflicts with coworkers, a lack of organizational support, a lack of recognition, adverse patient outcomes, and a stressed-out workplace (characterized by poor staffing, heavy workloads, and insufficient breaks) have all been associated with high work-related burnout (4, 5, 11). Whereas, having job autonomy, physical exercise, social support, leadership or superior support found to be protective against burnout (26). Preventive interventions are highly important to reduce the adverse effects of occupational stress and burnout, such as promoting self-care, social support, educational training, yoga and mindful interventions (27–29).

The intrinsic characteristics of the work performed by midwives, especially the strong emotional link with the women and the need to address highly complex situations, can lead to occupational stress. Previous studies show that personal variables (sociodemographic and personal related variables), professional/occupation-related and client related variables are all factors that can lead to occupational burnout (OB) in midwives (24, 25, 30–34) urnout not only affects physical and mental health but can also jeopardize the quality of care, as well as costs for the institution by increasing sick leave and rates of absenteeism. Consequently, in the World Health Organization classified burnout as an occupational disease and recognized it as an official medical diagnosis. To achieve universal health coverage around the world and highlight challenging conditions, the World Health Organization has designated as the year of the nurse and the midwife. High levels of anxiety, stress, and burnout high-light the need to find strategies to prevent and mitigate these problems. This effort could facilitate the growth of midwifery, improving the well-being and working conditions to break the burnout cycle in maternity services.

Even though various studies have proven that an increased burden of work-related burnout is a public health problem, little attention is given to the issue. Particularly, in developing nations, healthcare workers don’t receive enough physical and psychological care from the health management system. Additionally, there is a lack of data indicating the extent and factors related to work-related burnout among Ethiopian midwives. Generating evidence on the burden of the problem and associated factors is a vital step in mitigating the problem's far-reaching consequences of burnout, offers insights on focusing midwives’ mental health, and improves the quality of maternity care, ultimately helping to achieve the maternal, neonatal, and child health-related sustainable development goals (SDG). So, this study aimed to assess the magnitude of work-related burnout and associated factors among midwives working at public hospitals in northwest Ethiopia.

## Methods

### Study design and setting

A multicenter facility-based cross-sectional study was conducted from February 7 to April 30, 2022. The study involved midwives who worked in public hospitals in northwest Ethiopia. One of Ethiopia’s twelve regions is the regional state of Amhara. It’s situated in the northern and central regions of Ethiopia. Bahir Dar, the seat of the Amhara National Regional State, is situated 563 kilometers from Addis Ababa, the Ethiopian capital. It had 3,973 kebeles (493 urban kebeles), 15 zones, and 183 districts. As per the report from 2020, the total population of the region was 22,191,890. Additionally, there are 865 health centers, 60 primary hospitals, 13 general hospitals, and eight referral hospitals that provide healthcare services in the region (34). This study was carried out among midwives employed by public hospitals in the three randomly chosen zones of northwest Ethiopia. As a result, the study setting comprises all public hospitals in the zones of central Gondar, east Gojjam, and west Gojjam. In these randomly selected zones, there were 31 hospitals (four comprehensive specialized hospitals, two general hospitals, and 25 primary hospitals).

### Study population and eligibility criteria

The study participants were all midwives who were permanently employed in the maternity units of the randomly selected zones of the region. Thus, all midwives who were working in different obstetrics and gynecology wards with work experience of at least six months (35) and available during the data collection period were enrolled in the study. However, midwives who were temporarily employed or providing free service or experienced sorrow within two months of data collection were not included in the study.

### Sample size determination and sampling procedure

The sample size for this study was determined using the single population proportion formula by considering the 95% level of confidence, a proportion of work-related burnout 50% since there had been no prior study, and 5% of margin of error.

$n=\frac{{\left(Z\alpha /2\right)}^{2}P\left(1-p\right)}{d^{\;2}}$ Where n = required sample size, α = confidence level, Z = standard normal distribution curve value for 95% confidence level = 1.96, P = proportion of event, and d = margin of error.

Therefore, n = $\frac{{\left(1.96\right)}^{2}\;0.5\left(1-0.5\right)}{{\left(0.05\right)}^{2}}$ = 384. Finally, by considering a design effect of 1.5 and a non-response rate of 15%, the total sample size was 663. To select a sample of 663 midwives, stratification was done based on the level of public hospitals. In the selected public hospitals of northwest Ethiopia, 31 hospitals were identified, and the sample size was proportionally allocated depending on the level of hospitals. Then, the study participants were selected using a simple random sampling method. In the above hospitals, there were approximately 768 full-time working midwives at primary, general, and tertiary level of the hospitals (318, 50, and 400, respectively). Then, a final sample was selected from each stratum of hospital using the proportional to size allocation formula: Nf * ni /N, where: ni = number of midwives in each level of hospital, nf = final sample of the study, and N = total number of midwives in all hospitals. So, the proportional allocation of midwives for primary, general, and tertiary hospitals was 275, 43, and 345, respectively.

### Study variables and measurements

Work-related burnout (Low or High) was the dependent variable, whereas age, sex, religion, marital status, number of children, educational status, work experience, average monthly salary, level of health facility, working unit, perceived workload, work rotation, working hours per week, professional conflicts, coworker conflicts, experience poor obstetric outcomes or events, emotional demand of the job, stress, fairness in working area, intention to leave the profession, workplace violence, exposure to blood and body fluids or needle stick injuries, medical health problems, number of sick leaves per year, receive performance feedback, training, educational opportunities, participate in decision making, resource availability, co-worker support, superior support, recognition for work performance were independent variables of the study.

Work-related burnout was measured using seven items, each on a five-point Likert scale expressing the frequency or intensity related to the work of the participant. The responses were then rescaled into: 0, 25, 50, 75, and 100, as per the instructions given by the authors (2). Low work-related burnout is indicated when the mean score of work-related sub-domains is less than 50, whereas high work-related burnout is considered when the mean score of the work-related sub-domains is ≥50 (10, 36, 37). For descriptive purposes, work-related burnout is also defined as moderate (a mean score of 50–74), high (a score of 75–99), and severe (a score of 100) (4, 10).

Working hours per week are categorized as less than 40 h, 40–60 h, and greater than 60 h. If midwives’ working hours are greater than 60 h is considered “long working hours” (38). Emotional demand of the job is assessed using three questions of a five-point response and classified as high or low using the mean value as a cutoff point (39).

Coworker and superior support were assessed using three items with a five-point Likert scale for each variable and classified as poor or good based on the mean value as a cutoff point (39).

Workplace violence occurs when a midwife has experienced at least one form of violence, such as verbal and/or physical assault, at work in the previous year (40).

Verbal abuse: When a midwife cursed, yelled, threatened, or used a swear word. Behaviors that are humiliating, degrading, or otherwise show a lack of respect for a person’s worth and dignity (41). Additionally, physical violence was considered, if a midwife encountered any of the following: being assaulted by others through kicking, punching, slapping, stabbing, shooting, pushing, biting, spitting on, and/or pinching (41).

Stress status was measured using seven items with 0 to 3 responses on a Likert-scale and a total score greater than or equal to 15 was considered as having stress (42).

Intention to leave the profession: Three questions on a five-point response strongly disagree, disagree, neutral, agree, and strongly agree, was used to assess the midwives’ intention to leave their current position. It was measured as intended to leave, and not intended to leave, depending on the mean score (43).

### Data collection tool and quality assurance

A structured self-administered questionnaire of English version was used to collect data. The questionnaire consists of socio-demographic characteristics, work- and organizational-related variables prepared after reviewing pertinent literatures. Additionally, work-related burnout was assessed using seven items adopted from the Copenhagen Burnout Inventory. The tool consists of 19-items to measures burnout along the three domains: personal (six items), work-related (seven items), and client-related burnout (six items). The answers for the CBI items were given on a five-point Likert scale, ranging from 0 to 4, where 0 means never/a very low degree and 4 means always/to a very high degree. The answers were then transformed as follow: 0 = 0, 1 = 25, 2 = 50, 3 = 75, and 4 = 100, as per the instructions given by the authors of the tool. All items were positively skewed except for a single item, which needed a reverse scored. Thus, “Do you have enough energy for family and friends during leisure time?” The instrument is one of the standard tools that has been used and validated in various studies (2, 44, 45).

Six BSc midwives were recruited to collect data, whereas three MSc midwives were assigned for supervision. To assure the quality of the data, a one-day training was provided for data collectors and supervisors prior to the actual data collection. The training focused on the purpose of the study, the content of the questionnaires, and all the study protocols to be followed throughout the data collection process. Besides, to ensure that the data collecting tool was appropriate and understandable, a pretest was conducted at Debre Tabor Hospital on 5% of the estimated sample size. The completeness of the questionnaire was checked by the supervisors every day.

### Data management and analysis

After the data were checked for completeness, it was coded and entered into Epi-Data version 4.6 software, and exported to SPSS version 25 for further analysis. Both descriptive and analytic statistical procedures were carried out. Descriptive statistics such as means, medians, frequencies, proportions, tables, and figures were used to describe the characteristics of the study participants and display the study results. Following the descriptive analysis, a logistic regression model was fitted to assess the association between work-related burnout and independent variables. Bi-variable logistic regression was performed to identify candidate variables, and variables having a *p*-value of ≤ 0.2 were included in the multivariable logistic regression analysis model. In multivariable logistic regression analysis, variables with an adjusted odds ratio (AOR) with a *p*-value less than or equals to 0.05 at a 95% confidence interval (CI) were considered statistically significant predictors. Multi-collinearity between the independent variables was checked using the variance inflation factor (VIF), which indicates that there was no significant multi-collinearity since all variables have VIF <5. Finally, the goodness-of-fit of the model was also examined by Hosmer and Lemeshow and was found to be good.

## Results

### Socio-demographic characteristics

In this study, 663 participants were included, and 640 participants have provided a complete response, with a response rate of 96.53% (refusals and missed data). The median (± interquartile range) age of the participants was 28.5 ± 4.0 years, and more than half (52.7%) of the midwives were men. Regarding marital status, nearly two-third (63.1%) of them were married. Also, the median (± interquartile range) monthly salary was 10,390 birr with a 2494.5 IQR and the median (± interquartile range) year of experience in the profession was 5 ± 5 years (Table 1).

**Table 1:** Sociodemographic characteristics of midwives working at public hospitals in northwest Ethiopia, 2022 (*n* = 640).

Variables	Categories	Frequency (*n*)	Percent (%)
Age in years	20–30	479	74.84
	≥30	161	25.16
Sex of participant	Male	337	52.7
	Female	303	47.3
Participant’s religion	Christian	595	92.9
	Muslim	45	7.1
Marital status	Married	404	63.1
	Unmarried	246	36.9
Number of children	No child	196	30.6
	1–2 child	120	18.8
	≥ 3 children	324	50.6
Educational status	Diploma	104	16.3
	BSc degree	494	77.2
	MSc degree	42	6.5
Work experience	< 5 years	366	57.2
	5–10 years	198	30.9
	≥ 10 years	76	11.9

### Work-related variables

Nearly half of midwives (49.7%), were employed in Comprehensive Specialized hospitals, and about 276 (43.1%) of them worked at labor and delivery unit. Nearly two-thirds of the study participants (63.9%), reported the presence of a high workload at their workplace. Concerning workplace violence, about 359 (56.1%) of them faced verbal and/or physical violence from the patients, and/or their attendants at the workplace. Finally, nearly half (49.5%) of study participants reported as they experienced exposure of body fluid or sharp injury in the last 12 months and about 60.1% of them reported the presence of stress (See Table 2).

**Table 2:** Work-related variables among midwives working at public hospitals in northwest Ethiopia, 2022 (*n* = 640).

Variables	Category	Frequency (*n*)	Percent (%)
Level of hospital	Primary hospital	284	44.4
	General hospital	38	5.9
	Tertiary hospitals	318	49.7
Working unit	Gyn OPD	208	32.5
	Labor & delivery ward	298	46.6
	Gyn–OB inpatient ward	134	20.9
Job rotation	Every ≤6 months	376	58.8
	Every 12 months	122	19
	Every 24 months	142	22.2
Perceived high workload	Yes	580	90.6
	No	60	9.4
Faced poor obstetric outcomes in last one year	Yes	313	48.9
	No	327	50.1
Provider medical disorders	Yes	89	13.9
	No	551	86.1
Sick leave in the last one year	Yes	222	34.7
	No	418	65.3
Average working hours per week	< 40 h	207	32.3
	40–60 h	186	29.1
	> 60 h	247	38.6
Workplace violence in the last year	Yes	359	56.1
	No	281	43.9
Forms of violence (*N* = 359)	Verbal violence	278	77.4
	Physical violence	43	12
	Both	38	10.6
Experienced conflicts with colleagues	Yes	170	26.6
	No	470	73.4
Presence of professional conflicts	Yes	329	51.4
	No	311	48.6
Faced splash of body fluids/sharp injury	Yes	317	49.5
	No	323	50.5
Took post exposure prophylaxis (*N* = 317)	Yes	161	50.8
	No	156	49.2
Interest in midwifery profession	Yes	328	51.2
	No	312	48.8
Intention to leave a job	Yes	285	44.53
	No	355	55.47
Presence of stress	Yes	391	61.1
	No	249	38.9

*Gyn OPD, Gynecology outpatient department; Gyn-OB, gynecology and obstetrics.

### Health facility related factors

In terms of professional training and educational opportunities, the majority of respondents, 532 (83.1%) and 572 (89.4%), respectively, did not receive capacity-building training or educational opportunities to advance their careers. In addition to this, two-thirds (61.6%) of study participants stated receiving low superior support and majority of them (85.3%) did not receive recognitions for their work (Table 3).

**Table 3:** Health facility related factors among midwives working in public hospitals, northwest Ethiopia, 2022 (*n* = 640).

Professional training	Yes	108	16.9
	No	532	83.1
Educational opportunity	Yes	68	10.6
	No	572	89.4
Participation in decision Making	Low	405	63.3
	High	235	36.7
Received feedback for work performance	Yes	227	35.5
	No	413	64.5
Type of feedback	Oral	142	53.4
	Written	85	32.0
	Both	39	14.6
Received recognitions	Yes	94	14.7
	No	546	85.3
Superior support	Poor	394	61.6
	Good	246	38.4
Coworker support	Poor	319	49.8
	Good	321	50.2

### Magnitude and factors associated with work-related burnout

**Figure 1:**
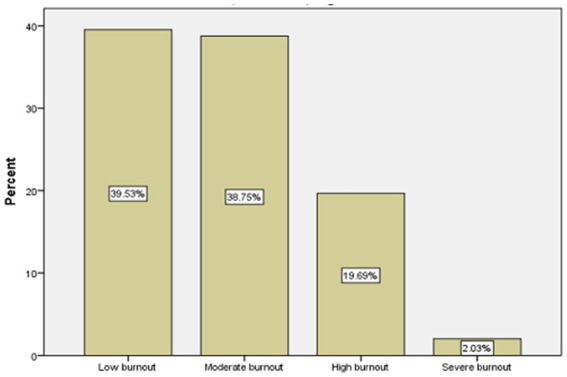
Level of work-related burnout according to CBI.

The prevalence of work-related burnout among midwives was found to be 60.47% (56.6, 64.2). The results of this study also showed that 38.75% of participants had moderate burnout, nearly 20% had high, and 2% had severe work-related burnout (See Figure 1).

To determine the factors influencing midwives’ work-related burnout, a bi-variable and multivariable logistic regression analysis was fitted. In the multivariable logistic regression analysis, job rotation every ≤ six months, working hours greater than 60 h per week, presence of workplace violence, exposure to blood and body fluids or needle stick injuries, high emotional demands of the job, and presence of stress were positively associated with work-related burnout, whereas having high superior support found to be protective of work-related burnout.

The odds of developing work-related burnout among midwives who experienced any form of workplace violence were 3.33 times (AOR = 3.33, 95% CI: 2.02, 5.48) greater than those who did not experience it. Study participants who worked greater than 60 h a week were 4.55 times more likely to have higher work-related burnout (AOR = 4.55, 95% CI: 2.78, 7.43) compared to those who worked less than 40 h per week. Also, the high emotional demanding nature of the profession (AOR = 8.85, 95% CI: 4.48, 17.47) increases the risk of having work-related burnout by 8.85. On the contrary, midwives who received good superior support (AOR = 0.38, 95% CI: 0.23, 0.63) had a 62% lower likelihood of experiencing work-related burnout as compared to their counterparts.

Furthermore, midwives who rotate from one working unit to another every less than or equals to six months were two times more likely to develop work-related burnout (AOR = 2.30, 95% CI: 1.28, 4.14) as compared to job rotation annually. Study participants who were exposed to blood and body fluids or needle stick injuries at the workplace had five times (AOR = 5.13, 95% CI: 3.12, 7.13) higher odds of having work-related burnout compared to their counterparts. Finally, the odds of having work-related burnout among midwives experiencing stress were found 2.63 times (AOR = 2.64, 95% CI: 1.63, 4.26) higher than those not stressed (Table 4).

**Table 4:** Bivariable and multivariable logistic regression analysis of factors associated with work-related burnout among midwives working at public hospital, northwest Ethiopia, 2022 (*n* = 640).

Predictor variables	WRB		Odds ratio at 95%	
	High	Low	COR	AOR
Sex of participant				
Male	225	112	1.75 (1.27, 2.41) **	1.35 (0.85,2.12)
Female	162	141	1	1
Job rotation				
Every ≤6 months	256	118	1.80 (1.18, 2.73) **	2.30 (1.28, 4.14) **
Every 24 months	55	67	0.64 (0.39, 1.04) *	1.24 (0.63, 2.43)
Every 12 months	62	80	1	1
Workplace violence				
Yes	225	61	4.37 (3.07,6.2) **	3.33 (2.02, 5.48) **
No	162	192	1	1
Working hours per week				
≤ 40 h	104	167	1	1
41–60 h	52	21	3.98 (2.26, 6.98) **	2.06 (1.02, 4.18) **
> 60 h	231	65	5.71 (3.95, 8.25) **	4.55 (2.78, 7.43) **
Conflict with colleagues				
Yes	137	33	3.65 (2.39, 5.57) **	1.17 (0.62, 2.20)
No	250	220	1	1
Professional conflicts				
Yes	239	90	2.93 (2.12, 4.06) **	1.36 (0.82, 2.26)
No	148	163	1	1
Coworker support				
Poor	214	105	1	1
Good	148	173	0.57 (0.42, 0.79) **	0.89 (0.55, 1.44)
Superior support				
Poor	273	121	1	1
Good	114	132	0.38 (0.28, 0.53) **	**0.38 (0.23, 0.63)** **
Blood & body fluid splash/ needle stick injuries				
Yes	228	89	2.64 (1.90, 3.67) **	5.13 (3.12, 7.13) **
No	159	164	1	1
Emotional demanding of the job				
High	156	13	12.47 (6.88, 22,58) **	8.85 (4.48, 12.35) **
Low	231	240	1	1
Stress level of participant				
Yes	269	122	2.45 (1.76, 3.40) **	2.64 (1.63, 4.26) **
No	118	131	**1**	**1**

* significant at *p*-value < 0.2, ** statistically significant at *p*-value < 0.05. Bold values indicates significantly associated factors.

### Discussion

This was a multicenter study that explore the prevalence work-related burnout and its contributing factors among midwives working at public hospitals in northwest Ethiopia. The prevalence of work-related burnout was found to be 60.47% (95% CI = 56.6–64.2). This finding was higher than a report at 55% in Senegal (46), 19.6% in Kenya (18), and 43.8% in Australia (47). The possible reasons for the differences could be related to the variation in study time, study settings, the variation of measuring tools and disparities in socioeconomic status and health systems. On the contrary, this finding was lower than a study conducted in Lithuania with 70.1% (48), and 82.2% work-related burnout in Jordan using a similar measuring tool (11). The disparities could be attributed to variations in the maternity care system (obstetrician-led), midwives providing a large amount of care, but having limited autonomy in their practice and decision-making, and a shortage of midwives in Jordan (11). In Lithuania, the maternity care system transitioned from obstetrician-led to midwifery-led, which might result in increased workloads and responsibilities, extended professional autonomy and recognition in caring for normal childbirths may motivate and raise their sense of professional ownership, which may increase their physical and psychological exhaustion (48).

This study demonstrated that workplace violence was a highly significant predictor of work-related burnout. Thus, midwives who had experienced any form of workplace violence in the last 12 months had higher odds of developing work-related burnout than those who had not. This finding was consistent with studies conducted among midwives and healthcare providers in Ethiopia (49–51) and Western Canada (4). The likely explanation could be the fact that suffering workplace violence had a wide spectrum psychological effect (post-traumatic stress disorder, anxiety, depression, vulnerability, and sleep disturbance), physical, and social consequences that left midwives to hate their job and workplace, which increased the risk of work-related burnout. Consequently, it affects the health of the midwives’, quality of maternity care provided, and increase turnover intention. Therefore, it’s important to offer training on conflict resolution strategies, cohesion of the staff, developing legislations for judicial punishment for committers. It’s also good to provide legal protection against any form of work place violence.

Work-related burnout is also significantly influenced by working hours greater than 60 h per week, as supported by different studies (38, 52, 53). This might be explained as prolonged working hours could be associated with job stress, sleep disturbance, exhaustion, and fatigue, which eventually leads to work-related burnout. Similarly, the more emotionally demanding nature of the midwifery profession was strongly associated with WRB, and this finding was in agreement with other studies (7, 38). The most likely explanation would be the emotionally and physically demanding nature of the midwifery profession, particularly managing obstetric and gynecologic complications, excessive workloads, and experiencing unfavorable maternal and neonatal outcomes, which requires midwives’ deep emotional and physical competence. Therefore, work-related burnout can be reduced through interventions such as reduced working hours, sufficient sleeping hours, regular shift work, work stress management, and mindfulness training.

Additionally, exposure to blood and body fluids or needle stick injuries was the other job-related variable that predisposed to WRB. This result was consistent with the findings of a study conducted among midwives and nurses in Ethiopia (49, 54). The plausible explanation could be related to worry about acquiring highly contagious infections such as the hepatitis B virus, HIV/AIDS, tuberculosis, and other infectious agents. Consequently, this exposure causes physical injury and psychological stress disorders, which affect the providers’ work performance, and quality of life. Midwives are a frontline professional that provide a comprehensive maternity care, and they are often exposed to biological and occupational hazards. So, the professionals should adhere to the standards of precautions, and health managers need to promote occupational health and safety. It’s also vital to increase uptake of vaccine hepatitis B vaccine, and making post exposure prophylaxis for HIV accessible to minimize emotional distress in case of accidental exposure.

This study also revealed that having good superior support is protective of work-related burnout, and according to different studies, supportive support or competent leadership is crucial in the health system to reduce job burnout (10, 55). The possible explanation might be the fact that supportive management enhances good communication, encourages involvement, fosters harmonious working environment, listen to their concerns, and find solutions to reduce job-related overwhelming conditions, which ultimately neutralize high work-related burnout. It also enhances the wellbeing of midwife professionals and enables the provision of quality maternal and newborn care.

WRB was also found to be higher among midwives who experience stress as compared to their counterparts, and this result was similar with other studies (8, 48). This could be related to a stressful working environment, and work-life imbalance leads to physical and emotional exhaustion. Finally, midwives with job rotation every less than or equals six months were more likely to have work-related burnout as compared to those with job rotation annually. This result was supported by the finding of previous study in nurse professionals (59). The possible explanation could be related to fewer staff, frequent rotation, low adaptation, and less stability in a specific working unit over a period of time.

## Strengths and limitations of the study

This study was a multi-centered institutional-based study with a relatively adequate sample size, for a better representation and generalizability. The present study also used the Copenhagen burnout inventory tool, which was an easy, flexible, and commercially free instrument to measure burnout worldwide. The authors strongly believe that the current study provides baseline evidence on the prevalence and its associated factors of work-related burnout in midwives. This enables health managers and other stakeholders to design effective preventive strategies against work-related burnout, which ultimately improves the health of midwives and the excellence of maternity care. However, this study was not without any limitations. Firstly, our study did not include midwives who were working at health centers, private hospitals, and clinics. Secondly, due to the cross-sectional nature, it’s makes difficult to determine causal relationships between the outcome and predictor variables. A longitudinal study might be more suitable because burnout is suggested to be a sequential process that develops and progresses through time.

## Conclusion and recommendations

In comparison to other studies, this study revealed that the burden of work-related burnout among midwives was relatively significant. Experiencing workplace violence, a job rotation of less than or equals to six months, long working hours greater than 60 h a week, high emotional demand of the profession, exposure to blood and body fluids or needle stick injuries, and stress were predictors that positively associated with work-related burnout, whereas receiving good superior support was negatively associated with work-related burnout. Thus, effective stress prevention strategies, the availability of personal protective equipment, adherence to universal precautions and safety, reduced working hours, supportive management, and legal protection against any form of workplace violence are critical for reducing work-related burnout, improving maternity care, and promoting the sustainability of the midwifery profession.

## Data availability statement

The original contributions presented in the study are included in the article/supplementary material, further inquiries can be directed to the corresponding author.

## Ethics statement

The studies involving humans were approved by University of Gondar Institutional Ethical Review Board. The studies were conducted in accordance with the local legislation and institutional requirements. The participants provided their written informed consent to participate in this study.

## Author contributions

BM: Conceptualization, Data curation, Formal analysis, Investigation, Methodology, Software, Supervision, Writing – original draft, Writing – review & editing. ZA: Conceptualization, Methodology, Writing – review & editing. TH: Conceptualization, Investigation, Methodology, Writing – review & editing. SA: Formal analysis, Investigation, Methodology, Supervision, Writing – review & editing. MA: Methodology, Writing – original draft. ET: Investigation, Methodology, Software, Supervision, Writing – review & editing. HA: Formal analysis, Methodology, Writing – review & editing. MD: Investigation, Methodology, Writing – original draft, Writing – review & editing. MM: Formal analysis, Investigation, Methodology, Writing – review & editing. NT: Conceptualization, Data curation, Formal analysis, Investigation, Methodology, Writing – original draft, Writing – review & editing. GA: Formal analysis, Investigation, Methodology, Software, Supervision, Writing – original draft, Writing – review & editing.
